# Prognostic value of pretreatment peripheral blood biomarkers in patients with head and neck squamous cell carcinoma treated with chemo/bioradiotherapy

**DOI:** 10.1007/s12094-025-03897-y

**Published:** 2025-05-05

**Authors:** Aina Sansa, Rosselin Vásquez, Cristina Valero, Cristina Vázquez, Anna Holgado, Julia Gayà, David Rubio, Xavier León

**Affiliations:** 1 Otorhinolaryngology – Head and Neck Surgery Department, Hospital Parc Taulí, Universitat Autònoma de Barcelona, Sabadell, Spain; 2 Otorhinolaryngology – Head and Neck Surgery Department, Hospital de La Santa Creu I Sant Pau, Universitat Autònoma de Barcelona, 90 Mas Casanovas Street, Barcelona, Spain; 3 Centro de Investigación Biomédica en Red de Bioingeniería, Biomateriales y Nanomedicina (CIBER-BBN), Madrid, Spain

**Keywords:** Biomarkers, Head and neck squamous cell carcinoma, Peripheral blood, Host, H-index

## Abstract

**Purpose:**

Hematological parameters obtained from a pretreatment peripheral blood lab test, as well as indices calculated from these parameters, are associated with the prognosis of the disease in patients with head and neck squamous cell carcinomas (HNSCC). The aim of this study is to determine which of the parameters or indices would have the best prognostic ability in HNSCC patients treated with chemo-radiotherapy or bio-radiotherapy.

**Methods/patients:**

Retrospective study of 345 patients with HNSCC treated with chemo/bio-radiotherapy, for whom a pretreatment lab test was available.

**Results:**

Of the parameters and indices analyzed, the one with the best prognostic capacity was the Host-index (H-index), which combines the prognostic capacity of hemoglobin and albumin levels, along with the absolute counts of neutrophils, monocytes, and lymphocytes. This index was available for 309 patients. Based on a recursive partitioning analysis, three groups of patients were defined according to the H-index. Considering as reference the patients with an H-index lower than 1.88 (*n* = 80, 25.9%), patients with an H-index value between 1.88 and 3.62 (*n* = 115, 37.2%) had a 2.74 times higher risk of dying due to the tumor (95% CI 1.41–5.30, *P* = 0.003), and patients with an H-index value greater than 3.62 (*n* = 114, 36.9%) had a 5.62 times higher risk (95% CI 2.95–10.81, *P* = 0.0001).

**Conclusion:**

The index derived from peripheral blood parameters that showed the best prognostic capacity in patients with head and neck squamous cell carcinoma treated with chemo/bio-radiotherapy was the H-index.

**Supplementary Information:**

The online version contains supplementary material available at 10.1007/s12094-025-03897-y.

## Introduction

For patients with head and neck squamous cell carcinoma (HNSCC), the current TNM classification system is based on the extent of the tumor at the local, regional, and distant levels. However, it has been shown that patient characteristics such as age, comorbidities, or general health status have prognostic value [1, 2]. Including additional information related to patient characteristics in classification systems could improve their prognostic accuracy and help individualize treatment plans.

Almost all patients with HNSCC have a blood test available at the time of tumor diagnosis. The results of the blood test can provide information about the pro-inflammatory state induced by the tumor through the absolute count of neutrophils, monocytes, and platelets, or plasma levels of acute-phase reactants such as C-reactive protein. Additionally, the lymphocyte count could act as a surrogate of the patient's immune system. Hemoglobin concentration has been interpreted as a surrogate marker of general health, with reduced levels being widely associated with worsened prognosis in patients with solid tumors, including HNSCC. Finally, total protein or albumin levels provide information regarding nutritional status, with reduced levels being associated with poorer oncological outcomes. In an attempt to increase the prognostic capacity of the information provided by peripheral blood parameters, several indices have been defined combining these individual parameters (reviewed in Valero et al. [3]).

Chemoradiotherapy is indicated for the treatment of advanced-stage HNSCC that is not amenable to surgical treatment or in cases where surgical treatment would cause significant functional impairment [4]. Several studies have analyzed the prognostic value of pretreatment peripheral blood biomarkers in patients treated with chemoradiotherapy, finding a significant correlation with individual parameters such as hemoglobin [5, 6], or with the combination of several parameters such as the Neutrophil-to-Lymphocyte Ratio (NLR) [6–8] or the Lymphocyte-to-Monocyte Ratio (LMR) [9]. To our knowledge, no studies have systematically analyzed the different hematological parameters and the indices derived from these parameters in HNSCC patients treated with chemoradiotherapy.

The aim of this study is to determine the prognostic capacity of pretreatment peripheral blood parameters in patients with HNSCC treated with chemo/bio-radiotherapy and to identify the parameter or index with the best prognostic capacity.

## Material and methods

This study was conducted retrospectively using information collected prospectively from a database of patients with head and neck malignant tumors diagnosed and treated at our center since 1985. Patients with squamous cell carcinoma located in the oral cavity, oropharynx, hypopharynx, or larynx, diagnosed between 2002 and 2020, without prior oncological treatment, treated with chemoradiotherapy or bioradiotherapy, who had a peripheral blood test performed within 4 weeks prior to the start of treatment, and with a follow-up of at least 3 years, were included in the study.

During the analyzed period, 367 patients with HNSCC received treatment with chemoradiotherapy/bioradiotherapy. Eighteen patients were excluded due to the absence of an appropriate peripheral blood lab test, and 4 patients were excluded for not completing the minimum follow-up period. The final cohort consisted of 345 patients.

Given the interaction between tobacco and alcohol consumption, a combined variable for toxic substance use was created with three categories: no consumption; moderate consumption (< 20 cigarettes/day and/or < 80 g alcohol/day); and severe consumption (≥ 20 cigarettes/day or ≥ 80 g alcohol/day). Staging was performed according to the current TNM edition at the time of tumor diagnosis.

For patients with oropharyngeal carcinomas, the Human Papillomavirus (HPV) status was determined by analyzing the presence of HPV DNA using RT-PCR techniques (LiPA25 v1 or CLART HPV 2). For all HPV DNA-positive samples, the immunohistochemical expression of p16INK4a was evaluated. The intensity of nuclear and cytoplasmic staining was determined, with specimens showing intense and diffuse staining of more than 70% of the tumor tissue considered p16INK4a positive. Tumors with both viral DNA presence and immunopositivity for p16INK4a were considered HPV-related (HPV-positive).

All patients were evaluated by the Tumor Board at our center, and treatment was proposed according to institutional protocols. Ten patients with oral cavity carcinomas received non-surgical treatment with chemo-radiotherapy (*n* = 6) or bio-radiotherapy (*n* = 4). Eight patients had locally advanced tumors (cT4b) and were not considered candidates for surgical treatment by the Tumor Board of our center, and the remaining 2 patients refused surgery. Radiotherapy treatment consisted of administering a dose of 70 Gy to the primary tumor and morphologically and/or metabolically positive lymph nodes, and 50 Gy to areas with a risk of microscopic disease, according to international consensus guidelines. Most patients received standard fractionation (2 Gy per fraction, 1 fraction/day, 5 days/week). Fourteen patients received hyperfractionated radiation (1.2 Gy per fraction, 2 fractions/day, 5 days/week). Chemotherapy treatment consisted of two to three cycles of cisplatin at a dose of 100 mg/m^2^ every 21 days (*n* = 225) or weekly carboplatin at a dose of 1.5 AUC (*n* = 59), initiated concurrently with radiotherapy. Patients treated with bioradiotherapy (*n* = 61) received 400 mg/m^2^ cetuximab the week before radiation therapy initiation and 250 mg/m^2^ cetuximab weekly during radiotherapy. A cervical lymph node dissection was included in the treatment regimen for 69 patients, performed after completing chemoradiotherapy (61 patients) or bioradiotherapy (8 patients).

The variables included in the study were plasma concentrations of glucose, urea, creatinine, aspartate aminotransferase (AST), alanine aminotransferase (ALT), albumin, and hemoglobin (Hb); the red cell blood distribution width (RDW); and absolute counts of neutrophils, eosinophils, basophils, monocytes, lymphocytes, and platelets. Additionally, several indices derived from the combination of analytical parameters, which have been associated with the prognosis of patients with HNSCC in the literature, were calculated and are shown in Table 1. The prognostic capacity of the peripheral blood parameters/indices was determined considering disease-specific mortality as the dependent variable using ROC curves. For parameters and indices in that an increase in the value of the variable was associated with better survival (urea, creatinine, AST, ALT, albumin, hemoglobin; absolute eosinophil, basophil, and lymphocyte counts; Prognostic Nutritional Index (PNI) and Hemoglobin-to-RDW Ratio (Hb-RDW), disease control was used as the dependent variable.

**Table 1:** Indices derived from the combination of analytical parameters ([absolute value x10E9/L]; albumin g/dL; hemoglobin g/dL; RDW: red cell distribution width)

Index	Formula
Neutrophil-to-lymphocyte ratio (NLR)	${\text{NLR}} = \frac{{\left[ {{\text{neutrophils}}} \right]}}{{\left[ {{\text{lymphocytes}}} \right]}}$
Platelet-to-lymphocyte ratio (PLR)	${\text{PLR}} = \frac{{\left[ {{\text{platelets}}} \right]}}{{\left[ {{\text{lymphocytes}}} \right]}}$
Monocyte-to-lymphocyte ratio (MLR)	${\text{MLR}} = \frac{{\left[ {{\text{monocytes}}} \right]}}{{\left[ {{\text{lymphocytes}}} \right]}}$
Systemic Inflammation Response Index (SIRI)	${\text{SIRI}} = \frac{{\left[ {{\text{neutrophils}}} \right]{ } \times { }\left[ {{\text{monocytes}}} \right]}}{{\left[ {{\text{lymphocytes}}} \right]}}$
Systemic Immune-Inflammation Index (SII)	${\text{SII}} = \frac{{\left[ {{\text{neutrophils}}} \right]{ } \times { }\left[ {{\text{platelets}}} \right]}}{{\left[ {{\text{lymphocytes}}} \right]}}$
Pan-immune-imflammation value (PIV)	${\text{PIV}} = \frac{{\left[ {{\text{neutrophils}}} \right]{ } \times { }\left[ {{\text{monocytes}}} \right]{ } \times { }\left[ {{\text{platelets}}} \right]}}{{\left[ {{\text{lymphocytes}}} \right]}}$
Prognostic Nutritional Index (PNI)	${\text{PNI}} = \left( {10{\text{ x albumin}}} \right) + (0.005{ } \times { }\left[ {{\text{lymphocytes}}} \right]$)
Host Index (H-index)	${\text{H - index}} = \left( {\frac{{\left[ {{\text{neutrophils}}} \right]{ } \times { }\left[ {{\text{monocytes}}} \right]}}{{{\text{albumin }} \times {\text{ hemoglobin }} \times { }\left[ {{\text{lymphocytes}}} \right]}}} \right) \times 100$
De Ritis ratio (DRR)	${\text{DRR}} = \frac{{{\text{AST}}}}{{{\text{ALT}}}}$
Hemoglobin-to-RDW ratio (Hb–RDW)	${\text{Hb}} - {\text{RDW}} = \frac{{{\text{hemoglobin}}}}{{{\text{RDW}}}}$

Next, we selected the Host Index (H-index), the biomarker that showed the best prognostic capacity based on the results of the ROC curves, and performed a recursive partition analysis with this variable, considering disease-specific survival (DSS) as the dependent variable. DSS was defined as the time from diagnosis to death attributed specifically to the HNSCC, with patients who died from other causes censored at the time of death. DSS corresponding to the categories defined by the recursive partition analysis was analyzed. Finally, a multivariable analysis was conducted, including DSS as the dependent variable, and tumor location, stage, treatment type, and H-index categories obtained from the recursive partition analysis as independent variables.

The relationship between qualitative and continuous variables was analyzed using the Student's *t*-test or ANOVA depending on the variables. The recursive partition analysis was performed using the CRT (Classification and Regression-Tree) method. Survival estimation was performed using the Kaplan–Meier method, comparing survival curves with the log-rank test. For multivariable analysis, the Cox proportional hazards model was used.

The study was approved by the Institutional Review Board of the center (IIBSP-CCC-2023-88) and was conducted in accordance with the principles outlined in the Declaration of Helsinki.

## Results

Table 2 shows the characteristics of the patients included in the study. During the follow-up period, 96 patients (27.8%) experienced a local tumor recurrence, 63 (18.3%) a regional recurrence, and 52 (15.1%) the appearance of distant metastases. A total of 121 patients (35.1%) died as a result of tumor progression. Table 1 in the Supplementary Material shows the location and tumor stage of patients who had a local (rT), regional (rN) or distant (rM) tumor recurrence, as well as those who died as a consequence of tumor progression (disease-specific death).

**Table 2:** Characteristics of the patients included in the study (*n* = 345)

	*N* (%)
Age	
< 50 years	32 (9.3%)
50–60 years	113 (32.8%)
60–70 years	117 (33.9%)
> 70 years	83 (24.1%)
Sex	
Male	281 (81.4%)
Female	64 (18.6%)
Toxics consumption	
No	36 (10.4%)
Moderate	47 (13.6%)
Severe	262 (75.9%)
Location	
Oral cavity	10 (2.9%)
Oropharynx	199 (57.7%)
Hypopharynx	52 (15.1%)
Larynx	84 (24.3%)
Local tumor extensión	
cT1	27 (7.8%)
cT2	116 (33.6%)
cT3	149 (43.2%)
cT4	53 (15.4%)
Regional tumor extension	
cN0	83 (24.1%)
cN1	58 (163.8%)
cN2	173 (50.1%)
cN3	31 (9.0%)
Stage	
II	10 (2.9%)
III	105 (30.4%)
IV	230 (66.7%)
Grade	
Well differentiated	16 (4.6%)
Moderately differentiated	272 (78.8%)
Poorly differentiated	57 (16.5%)
Treatment	
Chemoradiotherapy	284 (82.3%)
Bioradiotherapy	61 (17.7%)

Table 2 in the Supplementary Material shows the mean values of the analytical parameters and the indices derived from these parameters based on DSS. Patients who died due to the tumor had significantly higher values of alkaline phosphatase, neutrophils, monocytes, platelets, NLR, Platelet-to-Lymphocyte Ratio (PLR), Monocyte-to-Lymphocyte Ratio (MLR), Systemic Inflammation Response Index (SIRI), Systemic Immune-Inflammation Index (SII), Pan-Immune-Inflammation Value (PIV), and H-index than those in whom chemoradiotherapy/bio-radiotherapy treatment achieved disease control. Conversely, patients who died due to the tumor had significantly lower values of albumin, hemoglobin, lymphocytes, PNI, and Hb-RDW.

Table 3 shows the values of the area under the curve (AUC) obtained by analyzing the prognostic capacity of the different parameters and indices using ROC curves. Not all patients had information for all parameters. The table indicates the number of patients analyzed for each of the parameters and indices assessed. The parameter with the highest area under the curve was the H-index, an index that includes parameters in the numerator whose elevation is related to worse prognosis, such as elevated neutrophil and monocyte counts, and in the denominator, parameters whose decrease is related to worse prognosis, such as albumin and hemoglobin concentrations and lymphocyte count. A higher H-index value was associated with worse oncological outcomes.

**Table 3:** Results of the ROC curves for each of the parameters and indices analyzed

	*I*	AUC	95% CI	*P*
Glucose	329	0.509	0.416–0.597	0.886
Urea	337	0.529	0.459–0.599	0.415
Creatinine	343	0.523	0.450–0.595	0.524
AST	323	0.584	0.513–0.654	0.019
ALT	324	0.581	0.512–0.650	0.022
Alkaline phosphatase	315	0.589	0.500–0.678	0.047
Albumin	318	0.597	0.527–0.667	0.006
Hemoglobin	343	0.647	0.579–0.714	0.0001
RDW	343	0.578	0.493–0.664	0.080
Neutrophils	333	0.622	0.537–0.708	0.006
Eosinophils	333	0.515	0.444–0.585	0.683
Basophils	333	0.544	0.474–0.614	0.215
Monocytes	333	0.613	0.527–0.700	0.011
Lymphocytes	333	0.584	0.515–0652	0.019
Platelets	343	0.611	0.527–0.695	0.013
Neutrophil-to-lymphocyte ratio (NLR)	333	0.649	0.566–0.732	0.001
Platelet-to-lymphocyte ratio (PLR)	333	0.679	0.598–0.760	0.0001
Monocyte-to-lymphocyte ratio (MLR)	333	0.690	0.611–0.770	0.0001
Systemic Inflammation Response Index (SIRI)	333	0.672	0.592–0.753	0.0001
Systemic Immune-Inflammation Index (SII)	333	0.683	0.605–0.762	0.0001
Pan-immune-imflammation value (PIV)	333	0.685	0.607–0.763	0.0001
Prognostic Nutritional Index (PNI)	309	0.598	0.528–0.668	0.006
Host Index (H-index)	309	0.708	0.631–0.784	0.0001
De Ritis ratio (DRR)	322	0.538	0.451–0.626	0.393
Hemoglobin-to-RDW ratio (Hb–RDW)	343	0.608	0.539–0.678	0.002

Table 3 in the Supplementary Material shows the mean value and standard deviation of the H-index based on multiple clinical variables. Patients with no history of toxic substance use had significantly lower H-index values. A significant increase in the H-index value was observed as the category of local tumor extension (cT) increased, while no significant differences were observed based on regional extension (cN). No significant differences in the H-index were observed based on age, sex, primary tumor location, tumor grade, or treatment type. Information on HPV status was available for 138 oropharyngeal carcinoma patients, of whom 41 (29.7%) were classified as HPV-positive. For oropharyngeal carcinomas, HPV-positive patients had a significantly lower H-index value (*P* = 0.004).

**Fig. 1:**
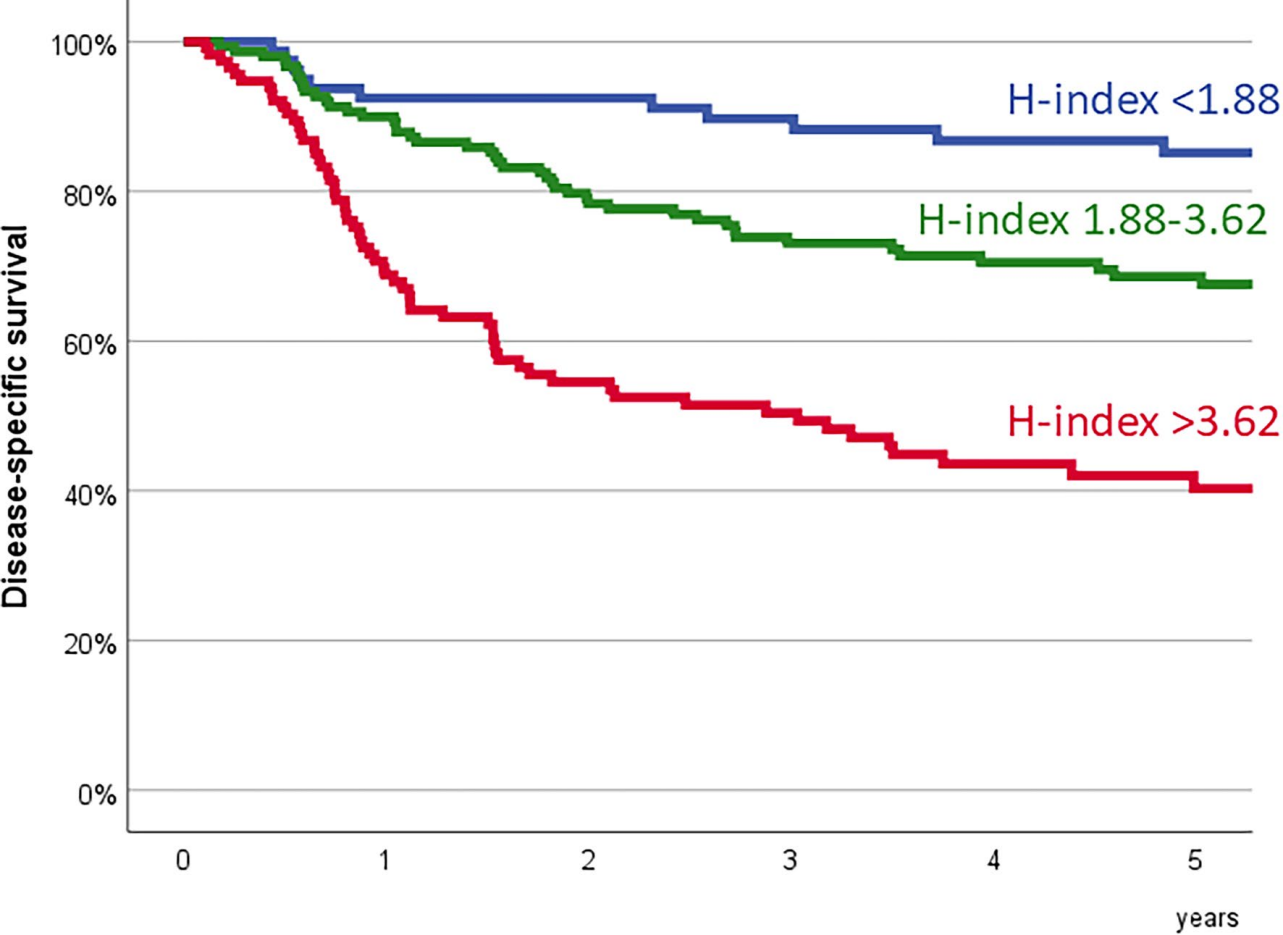
Disease-specific survival based on the H-index categories

Considering DSS as the dependent variable, recursive partition analysis defined three patient categories based on the H-index, with cutoff points at 1.88 and 3.62. Patients with an H-index lower than 1.88 (*n* = 80, 25.9%) had a 5-year DSS of 85.2% (95% CI 77.0–93.4%); patients with an H-index between 1.88 and 3.62 (*n* = 115, 37.2%) had a 5-year DSS of 68.6% (95% CI 60.8–76.4%); and patients with an H-index higher than 3.62 (*n* = 114, 36.9%) had a 5-year DSS of 40.3% (95% CI 30.3–50.3%). Figure 1 shows the survival curves based on the H-index categories (*P* = 0.0001).

Table 4 shows the 5-year DSS depending on the H-index category based on variables such as tumor location, stage, or treatment type. For patients with oropharyngeal carcinomas, DSS was evaluated based on HPV status. An ordered and significant decrease in survival was observed as the H-index category increased for nearly all analyzed variables, although in some cases, the differences did not reach statistical significance, such as for stage II tumors, hypopharyngeal tumors or HPV-positive oropharyngeal tumors.

**Table 4:** Five-year disease-specific survival (DSS) depending on the category of H-index based on the primary tumor location, staging, treatment type, and HPV status in patients with oropharyngeal carcinoma (*n* = 309)

	H-index	5-year DSS (95% CI)	*P*
Location			
Oral cavity	< 1.88	100%	0.012
	1.88–3.62	25.0% (0–67.5%)	
	> 3.62	50.0% (0.0–100%)	
Oropharynx	< 1.88	83.7% (72.5–94.9%)	0.0001
	1.88–3.62	66.2% (55.4–77.0%)	
	> 3.62	38.3% (26.3–50.3%)	
Hypopharynx	< 1.88	66.6% (39.6–93.6%)	0.599
	1.88–3.62	62.0% (41.2–82.8%)	
	> 3.62	50.4% (25.9–74.9%)	
Larynx	< 1.88	100%	0.0001
	1.88–3.62	69.5% (52.8–86.2%)	
	> 3.62	27.1% (0.0–54.3%)	
Stage			
II	< 1.88	100%	0.368
	1.88–3.62	66.7% (13.4–100%)	
	> 3.62	100%	
III	< 1.88	94.1% (82.6–100%)	0.017
	1.88–3.62	78.5% (66.0–91.0%)	
	> 3.62	61.6% (40.8–82.4%)	
IV	< 1.88	81.1% (70.5–91.7%)	0.0001
	1.88–3.62	54.5% (43.3–65.7%)	
	> 3.62	31.7% (20.7–42.7%)	
Treatment			
Chemoradiotherapy	< 1.88	86.6% (78.4–94.8%)	0.0001
	1.88–3.62	69.0% (60.2–77.8%)	
	> 3.62	45.2% (33.8–56.6%)	
Bioradiotherapy	< 1.88	71.4% (37.9–100%)	0.006
	1.88–3.62	37.9% (11.8–64.0%)	
	> 3.62	15.5% (0.0–34.1%)	
HPV status^a^			
HPV-negative	< 1.88	84.6% (65.0–100%)	0.0001
	1.88–3.62	64.3% (47.8–80.8%)	
	> 3.62	34.7% (19.2–50.2%)	
HPV-positive	< 1.88	92.3% (77.8–100%)	0.197
	1.88–3.62	75.6% (57.0–94.2%)	
	> 3.62	60.0% (17.1–100%)	

^a^125 patients with oropharyngeal cancer

An ordered and significant decrease in local (*P* = 0.0001), regional (*P* = 0.002), and distant (*P* = 0.003) control was observed as the H-index category increased. Table 4 in the Supplementary Material shows the 5-year recurrence-free survival values for local, regional, and distant recurrence based on the H-index categories.

Table 5 shows the results of a multivariable analysis in which DSS was considered the dependent variable, and tumor location, stage, treatment type, and H-index categories obtained from recursive partition analysis were included as independent variables. The H-index category maintained its prognostic capacity in the multivariable study. Compared to patients with an H-index value lower than 1.88, those with an H-index between 1.88 and 3.62 had a 2.37 times higher risk of dying from the tumor (95% CI 1.18–4.72, *P* = 0.014), and those with an H-index higher than 3.62 had a 5.64 times higher risk (95% CI 2.95–10.81, *P* = 0.0001). Another variable with prognostic capacity in the multivariable study was treatment type. Patients treated with bioradiotherapy had a 2.19 times higher risk of tumor-related mortality than those treated with chemoradiotherapy (95% CI 1.42–3.37, *P* = 0.0001).

**Table 5:** Multivariable analysis considering disease-specific survival as the dependent variable (*n* = 309)

	HR (95% CI)	*P*
Location		
Larynx	Ref	
Oral cavity	1.07 (0.40–2.87)	0.890
Oropharynx	1.07 (0.65–1.75)	0.785
Hypopharynx	1.08 (0.58–2.04)	0.789
Stage		
II	Ref	
III	1.52 (0.20–11.55)	0.683
IV	4.26 (0.59–30.71)	0.150
Treatment		
Chemoradiotherapy	Ref	
Bioradiotherapy	2.17 (1.44–3.27)	0.0001
H-index		
< 1.88	Ref	
1.88–3.62	2.74 (1.41–5.30)	0.003
> 3.62	5.62 (2.95–10.81)	0.0001

## Discussion

According to the results obtained in our study, of all the parameters and indices analyzed, the one with the best prognostic capacity was the H-index. The H-index is an index defined by Valero et al. [10] in a cohort of patients with oral cavity carcinomas treated with surgery. It combines the prognostic capacity of circulating neutrophil and monocyte counts (surrogate markers of the pro-inflammatory state induced by the tumor that is associated with an increased aggressiveness and treatment resistance), with an assessment of the immune system's ability to control tumor growth, represented by the lymphocyte count. It also adds an indirect evaluation of nutritional status based on albumin concentration and hemoglobin levels, which can be considered a surrogate marker of overall health. In the specific case of patients treated with radiotherapy, a low hemoglobin concentration could induce a certain degree of hypoxia within the tumor microenvironment, promoting treatment resistance [11].

The prognostic capacity of H-index has been validated in patients with HNSCC with tumors in different locations treated with both surgery and radiotherapy [12], as well as in patients with laryngeal carcinomas treated surgically [13]. Moreover, a high H-index value has been associated with complications such as trismus and osteoradionecrosis in patients with nasopharyngeal carcinomas treated with chemoradiotherapy [14].

In our cohort of patients, we observed a progressive increase in the H-index value as the local extent of the tumor (cT) increased (*P* = 0.0001). However, no differences were observed based on regional extension (*P* = 0.9). No significant differences were found in the H-index based on variables such as age, sex, tumor location, or histological grade either. Nonetheless, we found that the other variable associated with the H-index value was the history of toxic substance use, with higher values observed in patients with such a history. Lastly, when analyzing oropharyngeal carcinoma patients, we found that the average H-index value for HPV-positive tumors was significantly lower. In oropharyngeal carcinomas, the group of patients without a history of toxic substance use was predominantly composed of HPV-positive patients (46.3% of HPV-positive patients without a history of use vs. 2.1% of HPV-negative patients, *P* = 0.0001). It remains to be determined whether the reduced H-index value is related to the absence of toxic substance use or the differential carcinogenesis mechanism and prognosis involved in HPV-positive tumors.

The prognostic capacity of the H-index remained independent of the tumor's primary location, stage, treatment type, or HPV status (in oropharyngeal carcinoma patients). For most of these variables, there was an ordered and significant decrease in DSS as the H-index category increased. A multivariable study confirmed the independent prognostic capacity of the H-index. Compared to patients in the lowest H-index category (< 1.88), those with intermediate values (1.88–3.62) had a 2.74 times higher risk of dying from the tumor, and patients in the highest category (> 3.62) had a 5.62 times higher risk. Additionally, we also observed that an increase in the H-index category was associated with an ordered and significant decrease in local, regional, and distant recurrence-free survival.

Hemoglobin had the highest area under the curve value among all the individual parameters analyzed. Several studies have pointed out the prognostic capacity of hemoglobin concentration in patients with SCC treated with chemoradiotherapy [5, 15]. Nonetheless, although the prognostic capacity of hemoglobin concentration stands out, according to our results, indices that combine several parameters generally had a better prognostic capacity than individual parameters.

Among the analyzed indices, the one that achieved the highest discriminative value after the H-index was the Monocyte-to-Lymphocyte (MLR) ratio. Several studies have shown the relationship between MLR values and survival in HNSCC patients treated with chemoradiotherapy [9, 16]. Other indices that combine three or more hematological parameters and have been linked to survival in HNSCC patients, such as the Systemic Immune-Inflammation Index (SII) [17] or the Pan-Immune-Inflammation Value (PIV) [18], also showed a high discriminative prognostic capacity in our cohort of patients treated with chemo/bioradiotherapy.

The most extensively studied index in HNSCC patients is undoubtedly the Neutrophil-to-Lymphocyte Ratio (NLR). Several meta-analyses have highlighted the prognostic capacity of this index in HNSCC patients [19, 20]. Specifically, for patients with HNSCC treated with chemoradiotherapy, several studies have found a significant relationship between elevated NLR values and decreased survival [6–8, 21, 22]. According to our results, the NLR value was significantly related to DSS, but its prognostic capacity was inferior to that of other peripheral blood indices.

Our study has some limitations associated with its retrospective nature. On one hand, we lacked information about comorbidities or current treatments that could alter patients' peripheral blood values. Moreover, the routine lab tests performed at our center did not include certain parameters that quantify the systemic inflammatory state, such as C-reactive protein, which has been shown to be related to survival in HNSCC patients in other studies. In addition, it covers a long period of time, during which advances in chemotherapy and radiotherapy treatments have taken place and could have influenced the results obtained.

Nonetheless, our results suggest that the H-index is a robust biomarker with prognostic discriminative ability in HNSCC treated with chemo/bioradiotherapy, independent of patient characteristics. All these results lead us to consider that the inclusion of a parameter like the H-index could complement the prognostic capacity of commonly used staging systems like the TNM, which are based solely on tumor’s anatomical extension.

## Conclusion

Certain peripheral blood parameters obtained from routine blood tests, as well as indices calculated from these parameters, have the prognostic capacity in patients with HNSCC treated with chemo/bioradiotherapy. Among the parameters and indices analyzed, the one with the best prognostic capacity was the H-index, an index combining the prognostic information provided by albumin and hemoglobin levels, and neutrophil, monocyte, and lymphocyte counts.

## Supplementary Information

Below is the link to the electronic supplementary material.

Supplementary file1 (DOCX 25 KB)

## Data Availability

The data that support the findings of this study are available from the corresponding author upon reasonable request.
