# Supplementary material for: Prognostic value of pretreatment peripheral blood biomarkers in patients with head and neck squamous cell carcinoma treated with chemo/bioradiotherapy
Source: Clin Transl Oncol. 2025 May 5;27(11):4242–50. doi: 10.1007/s12094-025-03897-y (PMC12559090; doi:10.1007/s12094-025-03897-y)
Supplement: Supplementary file 1 — Supplementary file1 (DOCX 25 KB) [file 12094_2025_3897_MOESM1_ESM.docx]

**SUPPLEMENTARY MATERIAL:**

**Prognostic value of pretreatment peripheral blood biomarkers in patients with head and neck squamous cell carcinoma treated with chemo/bioradiotherapy**

Aina Sansa^a^, Rosselin Vásquez^b^, Cristina Valero^b^, Cristina Vázquez^b^, Anna Holgado^b^, Julia Gayà^b^, David Rubio^b^, Xavier León^b,c,*^

^a^ Otorhinolaryngology – Head and Neck Surgery Department. Hospital Parc Taulí, Universitat Autònoma de Barcelona, Sabadell, Spain

^b^ Otorhinolaryngology – Head and Neck Surgery Department. Hospital de la Santa Creu i Sant Pau, Universitat Autònoma de Barcelona, Barcelona, Spain

^c^ Centro de Investigación Biomédica en Red de Bioingeniería, Biomateriales y Nanomedicina (CIBER-BBN), Madrid, Spain

***Corresponding author**: Dr. Xavier León

Otorhinolaryngology – Head and Neck Surgery

Hospital de la Santa Creu i Sant Pau

90 Mas Casanovas Street

08041 Barcelona, Spain

Phone: +34 935565679

Fax: +34 935565604

E-mail: xleon@santpau.cat

**Tabla 1.** Location and tumor stage of patients who had a local (rT), regional (rN) or distant (rM) tumor recurrence, as well as those who died as a consequence of tumor progression (disease-specific death; DSS).

|  |  | **rT** | **rN** | **rM** | **DSS** |
| --- | --- | --- | --- | --- | --- |
| **Location** | Oral cavity (n=10) | 7 (70.0%) | 2 (20%) | 0 (0%) | 5 (50%) |
|  | Oropharynx (n=199) | 48 (24.1%) | 36 (18.1%) | 29 (14.6%) | 73 (36.7%) |
|  | Hypopharynx (n=52) | 14 (26.9%) | 11 (21.2%) | 11 (21.2%) | 20 (38.5%) |
|  | Larynx (n=84) | 27 (32.1%) | 14 (16.7%) | 12 (14.3%) | 23 (27.4%) |
| **Stage** | II (n=10) | 2 (20.0%) | 1 (10.0%) | 0 (0%) | 1 (10.0%) |
|  | III (n=105) | 27 (25.7%) | 8 (7.6%) | 5 (4.8%) | 20 (19.0%) |
|  | IV (n=230) | 67 (29.1%) | 54 (23.5%) | 47 (20.4%) | 100 (43.5%) |

**Table 2.** Mean value (standard deviation) of the different analytical parameters and indices based on disease-specific survival.

|  | **Disease control** | **No disease control** | **P** |
| --- | --- | --- | --- |
| Glucose | 5.73 (1.23) | 5.90 (1.91) | 0.321 |
| Urea | 5.60 (2.06) | 5.72 (2.84) | 0.665 |
| Creatinine | 78.24 (21.06) | 79.56 (24.65) | 0.602 |
| AST | 23.62 (19.46) | 23.33 (18.48) | 0.899 |
| ALT | 24.29 (18.57) | 21.29 (16.82) | 0.153 |
| Alkaline phosphatase | 79.59 (25.27) | 88.50 (33.61) | 0.008 |
| Albumin | 42.73 (3.95) | 41.06 (4.54) | 0.001 |
| Hemoglobin | 14.25 (1.47) | 13.53 (1.27) | 0.0001 |
| RDW | 13.47 (1.11) | 13.68 (1.45) | 0.132 |
| Neutrophils | 5.15 (2.49) | 5.85 (2.55) | 0.016 |
| Eosinophils | 0.19 (0.14) | 0.19 (0.18) | 0.957 |
| Basophils | 0.04 (0.03) | 0.04 (0.02) | 0.259 |
| Monocytes | 0.70 (0.26) | 0.78 (0.26) | 0.004 |
| Lymphocytes | 2.18 (0.81) | 1.91 (0.72) | 0.003 |
| Platelets | 252.15 (82.39) | 281.61 (93.48) | 0.178 |
| Neutrophil-to-Lymphocyte Ratio (NLR) | 2.69 (1.78) | 3.80 (4.09) | 0.001 |
| Platelet-to-Lymphocyte Ratio (PLR) | 134.04 (96.78) | 172.45 (107.27) | 0.001 |
| Monocyte-to-Lymphocyte Ratio (MLR) | 0.36 (0.21) | 0.47 (0.26) | 0.0001 |
| Systemic Inflammation Response Index (SIRI) | 1.99 (1.814) | 3.15 (4.00) | 0.001 |
| Systemic Immune-Inflammation Index (SII) | 721.25 (725.2) | 1134.7 (1660.7) | 0.004 |
| Pan-Immune-Imflammation Value (PIV) | 556.64 (680.8) | 989.3 (1647.08) | 0.001 |
| Prognostic Nutritional Index (PNI) | 426.80 (39.68) | 410.56 (45.40) | 0.0001 |
| Host Index (HI) | 3.40 (3.15) | 5.55 (5.29) | 0.00001 |
| De Ritis Ratio (DRR) | 1.12 (0.49) | 1.23 (0.53) | 0.089 |
| Hemoglobin-to-RDW Ratio (Hb-RDW) | 10.65 (1.43) | 10.02 (1.80) | 0.001 |

**Table 3.** Mean value (standard deviation) of the H-index based on different clinical variables (n=309).

|  |  | **N** | **Host Index** | **P** |
| --- | --- | --- | --- | --- |
| Age | <50 years | 27 | 3.73 (2.90) | 0.729 |
|  | 50-60 years | 101 | 3.96 (5.02) |  |
|  | 60-70 years | 106 | 4.09 (3.31) |  |
|  | >70 years | 75 | 4.58 (4.27) |  |
| Sex | Male | 250 | 4.28 (4.26) | 0.206 |
|  | Female | 59 | 3.52 (3.49) |  |
| Toxics consumption | No | 34 | 2.76 (1.98) | 0.047 |
|  | Moderate | 41 | 3.49 (2.74) |  |
|  | Severe | 234 | 4.45 (4.51) |  |
| Location | Oral cavity | 10 | 5.30 (4.18) | 0.632 |
|  | Oropharynx | 180 | 4.25 (4.07) |  |
|  | Hypopharynx | 45 | 4.14 (5.54) |  |
|  | Larynx | 74 | 3.70 (3.22) |  |
| Local extension | cT1 | 21 | 2.02 (1.14) | 0.0001 |
|  | cT2 | 106 | 3.32 (2.99) |  |
|  | cT3 | 130 | 4.36 (4.41) |  |
|  | cT4 | 52 | 6.09 (5.27) |  |
| Regional extension | cN0 | 73 | 4.13 (3.88) | 0.900 |
|  | cN1 | 56 | 4.12 (3.55) |  |
|  | cN2 | 152 | 4.25 (4.74) |  |
|  | cN3 | 28 | 3.60 (1.80) |  |
| Stage | II | 9 | 2.50 (1.59) | 0.293 |
|  | III | 94 | 3.83 (3.19) |  |
|  | IV | 206 | 4.35 (4.55) |  |
| Grade | Well differentiated | 13 | 5.09 (3.73) | 0.695 |
|  | Moderately differentiated | 249 | 4.10 (4.27) |  |
|  | Poorly differentiated | 47 | 4.05 (3.48) |  |
| Treatment | Chemoradiotherapy | 253 | 3.97 (4.02) | 0.122 |
|  | Bioradiotherapy | 56 | 4.91 (4.58) |  |
| HPV* | Negative | 90 | 5.11 (4.69) | 0.004 |
|  | Positive | 35 | 2.65 (2.58) |  |

*125 patients with oropharyngeal cancer

**Table 4.**  Five-year recurrence-free survival (local, regional, and distant) based on the H-index categories (n=309).

|  | **H-index** | **5-year recurrence-free survival (95% CI)** | **P** |
| --- | --- | --- | --- |
| Local | <1.88 | 85.8 (78.0-93.6%) | 0.0001 |
|  | 1.88-3.62 | 71.1 (63.3-78.9%) |  |
|  | >3.62 | 55.6 (45.8-65.4%) |  |
| Regional | <1.88 | 87.9 (77.7-98.1%) | 0.002 |
|  | 1.88-3.62 | 79.7 (72.6-86.8%) |  |
|  | >3.62 | 70.8 (61.6-80.0%) |  |
| Distant | <1.88 | 92.4 (86.5-98.3%) | 0.003 |
|  | 1.88-3.62 | 82.1 (75.0-98.2%) |  |
|  | >3.62 | 72.5 (62.7-82.3%) |  |
